# Reconfigurable Intelligent Surface-Based Physical Layer Authentication Enhancement

**DOI:** 10.3390/s26134024

**Published:** 2026-06-24

**Authors:** Binting Su, He Fang, Junhui Zhao

**Affiliations:** 1 Network and Data Center, Fujian Normal University, Fuzhou 350117, China; bintingsu@fjnu.edu.cn; 2 College of Computer and Cyber Security, Fujian Normal University, Fuzhou 350117, China; 3 School of Electronics and Information Engineering, Beijing Jiaotong University, Beijing 100044, China; junhuizhao@bjtu.edu.cn

**Keywords:** physical layer authentication, reconfigurable intelligent surface, intelligent controllable reflection, watermark hopping, performance improvement

## Abstract

This article introduces the reconfigurable intelligent surface (RIS) to physical layer authentication (PLA) designs to explore the utility of RIS in both the radio frequency fingerprint (RFF)/channel fingerprint (CF)-based PLA technique and the tag embedding (TE)-based PLA technique. Two new PLA schemes are proposed, i.e., the controllable reflection-based PLA (CR-PLA) scheme and the watermark hopping-based PLA (WH-PLA) scheme, where the role of RIS is discussed and analyzed carefully. First of all, considering the performance of RFF/CF-based PLA technique is degraded by the inaccurate feature estimation, the CR-PLA scheme is proposed to improve the feature estimation accuracy and to amplify the estimation differences among multiple devices through reconfiguring the wireless propagation channel. Then, to improve the performance of the TE-based PLA technique and introduce it to the RIS-aided systems, the WH-PLA scheme is developed. This scheme adds the security information on the pilot signal or message signal alternatively for authentication according to a designed pseudorandom embedding sequence with high uncertainty and randomness. Our simulation results verify the better performance of the proposed schemes compared with the existing schemes. The challenges and open issues of PLA designs in the RIS-aided wireless communication systems are also presented.

## 1. Introduction

**Figure 1:**
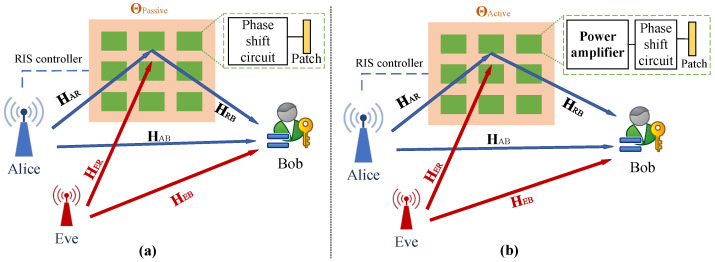
RIS-aided wireless communication systems: (**a**) passive system and (**b**) active system, where Alice and Bob are legitimate users and Eve is an attacker. Eve aims at imitating Alice to fool Bob.

The sixth generation (6G) systems are expected to further improve the reliability, capacity, power efficiency, and low latency trade-offs in support of challenging applications ranging from autonomous systems to extended reality [1]. Compared to the traditional wireless communications with complex radio frequency, RIS has emerged as one of the key technologies to reconfigure the wireless propagation environment via software-controlled reflection [2]. It can be used to increase spectrum and energy efficiency, to expand transmission distance and coverage area, and to support secure communication, as well as simultaneous power transfer and information transmission, just to name a few [2,3]. Figure 1 demonstrates the passive and active RIS-aided wireless communication systems and highlights their difference. The passive RIS reflects the impinging signal and intelligently adjusts the phase shifts to constructively superimpose and enhance the signal, while the appealing feature of the active RIS is that it can not only adjust the phase shifts but also amplify the received signal attenuated from the first hop to a normal strength level [4,5].

**Figure 2:**
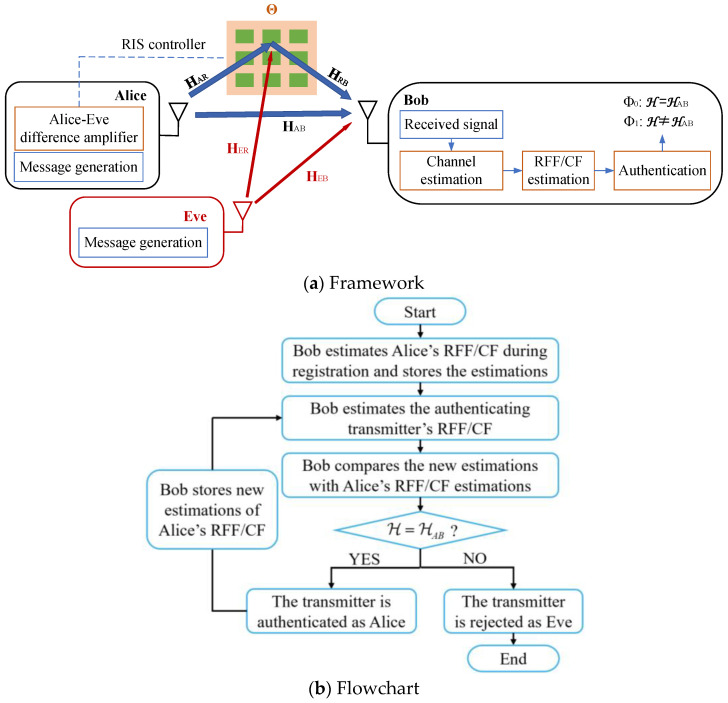
The proposed CR-PLA scheme. Bob identifies Alice from Eve based on the RFF/CF estimation. (**a**) Framework; (**b**) Flowchart.

As shown in Figure 1, the RIS-enabled systems suffer from the eavesdropping and spoofing attacks due to the open broadcast nature of radio signal propagation. Eve aims at eavesdropping on the communications between Alice and Bob, and then imitating Alice to cheat Bob for illegal advantages. The attacks will bring a number of security threats, such as unauthorized access, privacy leakage, and data injection, leading to enormous losses. The PLA techniques have been widely studied as promising alternative solutions for authenticating the devices/messages by leveraging the properties of the physical channel, which enhance security requiring for short time latency, low cost, and storage resources [6]. Figure 2 compares the existing PLA techniques as well as their advantages and challenges, including the RFF-based, CF-based, and TE-based techniques.

Table 1 provides comparisons of different PLA techniques. To be more specific, the RFF-based technique relies on the hardware features of devices for authentication, e.g., carrier frequency offsets (CFO) [7], in-phase/quadrature (I/Q) imbalance [8], and nonlinear characteristics caused by digital-to-analog converter, power amplifier, and so on. These RFFs are hardware-dependent and difficult for the other devices to clone. The main challenges of the RFF-based technique include the minor differences in RFF among multiple devices, and great difficulty in estimating accurate RFFs in the noisy wireless communication environment. The CF-based technique utilizes the features of communication channels for authentication, e.g., channel impulse response (CIR)/channel frequency response (CFR) [9], and received signal strength indicator (RSSI) [10]. Its performance suffers from the complex time-varying channels as well as depends on the location and physical environment of devices. The TE-based technique embeds security information (e.g., tag, fingerprint, and watermark) in the transmitted messages/signals at the physical layer for authentication, e.g., encodes pilot/message [11], multiplies tag in message [12], superimposes tag on message signal [13], and superimposes tag on pilot signal [14]. Although they provide high randomness and covertness, the signal-to-noise ratio (SNR) of the received signal and the spectral efficiency of the communication systems will be decreased.

### 1.1. Motivations

The RIS has been studied for the security enhancement at the physical layer, including the physical layer security scheme of [15], the physical layer key generation scheme of [16], and the PLA scheme of [17]. The work of [16] identifies static and wave-blockage environments as two RIS-empowered physical-layer key generation applications. Its experimental results in a static environment demonstrate that RIS can enhance the entropy of the secret key, achieving a key generation rate of 97.39 bit/s with a bit disagreement rate of 0.083. Moreover, the authors of [17] proposed challenge-response mechanisms in the context of physical layer security with the assistance of RIS. The verifier, instead of sending a challenge, changes the physical properties of the electromagnetic environment and expects to receive a properly modified signal from the device under verification.

When RIS meets PLA, two questions remain to be answered:

Q1. How to utilize the RIS for PLA enhancement?

As shown in Figure 2, the open issue of both RFF-based and CF-based PLA techniques is the improvement of feature estimation accuracy for identifying multiple devices, especially in the highly dynamic communication environments and the dense wireless communication systems [6,18]. It is mainly caused by the minor differences in RFF among multiple devices and the complex time-varying channels encountered. To improve the PLA performance, it is required to amplify the RFF estimation differences among different devices, or to increase the feature estimation accuracy in the noisy wireless communication environments. Hence, we introduce the RIS to the PLA technique through controlling the reflection and mitigating the interference of transmit signals to facilitate the estimation of RFFs and CFs.

Q2. How to design the PLA schemes in RIS-aided systems?

As shown in Figure 1, the composite channel from Alice to Bob through each element of the RIS is a concatenation of three components, namely for the Alice–RIS link, RIS’s reflection, and the RIS–Bob link, as shown in Figure 1. Such a composite channel brings great difficulty for PLA in designing and decoding the security information for achieving the best performance in both communication and security. Moreover, to improve the communication performance as well as to achieve better energy and spectrum efficiency, the RIS-aided systems require the channel estimates to set the reflection coefficients. This facilitates the PLA designs requiring channel estimate and message recovery, e.g., the TE-based PLA technique [11,12,13,14], but also brings higher requirement on designing the PLA schemes while guaranteeing the performance of RIS-aided communications. Hence, the PLA designs for RIS-aided systems should well consider both advantages and disadvantages of existing methods, and achieve better performance in authentication accuracy, covertness, robustness, and efficiency.

### 1.2. Contributions

In this article, two new PLA schemes are proposed to answer the above two questions, respectively. Firstly, we develop a controllable reflection-based PLA scheme by amplifying RFF/CF estimation differences between Alice and Eve when the signals are amplifying and/or reflecting at RIS, which is abbreviated to the CR-PLA scheme. Then, a watermark hopping-based PLA scheme is proposed for passive RIS-aided systems by embedding the authentication tag on pilot or message signals with high uncertainty and randomness, which is abbreviated to the WH-PLA scheme. The contributions of this article are summarized as follows:

(1) This article introduces RIS to the PLA designs for securing the wireless communications with short time latency and high robustness. The controllable reflection of RIS and physical layer properties are fully utilized in the proposed PLA schemes to achieve both communication improvement and security enhancement.
(2) We design the reflection coefficient of the RIS in the CR-PLA scheme to amplify the difference between Alice–Bob and Eve–Bob channels and to increase the RFF/CF estimation accuracy for authentication performance improvement. This scheme effectively suppresses the Eve–Bob channel leakage and enhances the physical layer security of the legitimate transmission link.
(3) The proposed WH-PLA scheme designs a pseudorandom embedding sequence for Alice to superpose the generated tag on the pilot signal and source message signal alternatively to increase the efficiency and randomness of authentication. This scheme jointly optimizes the security and communication performance by allocating the power factors of tag and pilot/message as well as by designing the reflection coefficient of RIS.

The rest of this paper is organized as follows. In Section 2, we present the system model and assumptions. In Section 3, we propose the CR-PLA scheme and WH-PLA scheme, and analyze their theoretical performance. The simulation verification and performance analysis of our schemes are given in Section 4. Section 5 presents challenges and open research issues. Finally, Section 6 concludes this paper. The symbols used in the paper are shown in Table 2.

## 2. System Model

A RIS-aided wireless communication system is shown in Figure 1, which is a single-input and single-output (SISO) system. This system suffers from spoofing attacks. The conventional Alice–Bob–Eve model is given, where Eve intercepts the wireless communications and imitates Alice to access the network/communicate with Bob, leading to the privacy leakage, data injection, and denial of service, just to name a few. Bob identifies Eve from Alice by utilizing the physical layer properties.

### 2.1. Channel Model

As shown in Figure 1, the channel gain of direct-link channel from Alice to Bob (i.e., Alice–Bob link) is denoted by $H_{\mathrm{AB}}$. The RIS-aided link is composed of a forward channel from Alice to the RIS (i.e., Alice–RIS link) and a backward channel from the RIS to Bob (i.e., RIS–Bob link). Their channel gains are denoted as $H_{\mathrm{AR}}$ and $H_{\mathrm{RB}}$, respectively. Similarly, we denote the channel gain of direct link from Eve to Bob (i.e., Eve–Bob link) as $H_{\mathrm{EB}}$ and the channel gain of forward channel from Eve to the RIS (i.e., Eve–RIS link) as $H_{\mathrm{ER}}$. Specifically, reflecting coefficient matrix of the RIS $\Theta =\mathrm{diag}(\phi_{1},\phi_{1},\cdots ,\phi_{N})$ is diagonal. The reflecting coefficient of the n-th reflection element at the active RIS is denoted by $\phi_{n},n=1,2,\ldots ,N$, where N is the number of reflection elements of the RIS.

All wireless channels are modeled as independent block-fading Rayleigh channels. The channel gains of direct channel $H_{\mathrm{AB}}$, $H_{\mathrm{EB}}$, and the RIS-related channels $H_{\mathrm{AR}}$, $H_{\mathrm{RB}}$, $H_{\mathrm{ER}}$ are complex Gaussian random variables with zero mean and unit variance for the small-scale fading component, i.e., $H_{\mathrm{AB}}∼N\left(0,\sigma_{\mathrm{AB}}^{2}\right)$, $H_{\mathrm{EB}}∼N\left(0,\sigma_{\mathrm{EB}}^{2}\right)$, $H_{\mathrm{AR}}∼N\left(0,\sigma_{\mathrm{AR}}^{2}\right)$, $H_{\mathrm{RB}}∼N\left(0,\sigma_{\mathrm{RB}}^{2}\right)$, and $H_{\mathrm{ER}}∼N\left(0,\sigma_{\mathrm{ER}}^{2}\right)$, where the variances incorporate large-scale path loss. The path loss is distance-dependent and follows the log-distance model $\mathrm{PL}\left(d\right)={\mathrm{PL}}_{0}+10\gamma \log_{10}\left(d/d_{0}\right)+X_{\sigma }$, where ${\mathrm{PL}}_{0}$ is the path loss at reference distance $d_{0}$, γ is the path loss exponent, and $X_{\sigma }∼N\left(0,\sigma_{\mathrm{SF}}^{2}\right)$ is shadow fading. The thermal noise at Bob and at the RIS (for active RIS case) is additive white Gaussian noise (AWGN) with zero mean and variance $\sigma_{\eta }^{2}$.

More specifically, some assumptions on the channel model are given as follows:

♦ All Alice–RIS and RIS–Bob distances exceed the Fraunhofer distance, allowing plane-wave approximation and independent phase shifts for beam steering.
♦ Legitimate parties (Alice and Bob) have perfect Channel State Information (CSI) of the Alice–Bob, Alice–RIS, and RIS–Bob links via pilot estimation, while Eve’s CSI is unknown.
♦ The RIS controller is connected to Alice via a reliable low-rate wired/wireless link. Alice updates the reflection coefficient matrix at the beginning of each coherence interval. The control link is assumed secure and error-free.
♦ Time and frequency synchronization is established between Alice and Bob prior to transmission. No eavesdropper can tamper with or intercept the control signaling.

### 2.2. Adversarial Model and Assumptions

In this paper, we present the adversarial model and assumptions as follows:

♦ Two devices communicate over the public channel and end-points are not trusted.
♦ Eve may impersonate Alice to either seek illegal benefits from the system/Bob, inject false data into the system/Bob, intercept sensitive messages, and execute other malicious behaviors.
♦ Eve is a single malicious device, able to perform any kind of signal processing techniques on its received signals.
♦ Eve is located at least half-wave length away from Alice.

To verify the transmitter, the PLA technique is used in the RIS-aided wireless communication system by superimposing carefully designed secret information on the transmitted waveforms, e.g., message or pilot signal.

## 3. Proposed Schemes

This article proposes two PLA schemes by fully utilizing both the controllable reflection of RIS and the physical layer superiority. The CR-PLA scheme is proposed based on the RIS, where the RFF/CF estimation differences between Alice and Eve are amplified by increasing the estimation accuracy. By superimposing carefully designed secret information on the waveforms, authentication is added to the signal at the physical layer in the WH-PLA scheme for the RIS-aided communication systems without requiring additional bandwidth, complex process, and long latency.

### 3.1. CR-PLA: Controllable Reflection-Based PLA Scheme

To answer question Q1, the CR-PLA scheme is firstly proposed in this article, where the RIS is utilized to increase the RFF/CF estimation accuracy and to amplify the differences in feature estimations between Alice and Eve, thus to improve the PLA performance. The proposed scheme is shown in Figure 2, where both the active RIS and passive RIS are considered to be applied.

*(1) Designs at Alice:* As shown in Figure 1b, the source signals traveling through the indirect link are amplified by the RIS in the active RIS-aided system. At the same time, the active RIS unintentionally amplifies the RIS-correlated noise. Hence, the signal that arrives at Bob can be expressed as

(1) $$R_{\mathrm{Active}}=\sqrt{p_{t}}H_{\mathrm{AB}}S+H_{\mathrm{RB}}\Theta_{\mathrm{Active}}(\sqrt{p_{t}}H_{\mathrm{AR}}S+\eta_{1})+\eta_{2},$$

where S is the transmit signal and $p_{t}$ represents the transmit power of Alice. $H_{\mathrm{AB}}$, $H_{\mathrm{AR}}$ and $H_{\mathrm{RB}}$ are the channel gain vectors of the Alice–Bob link, Alice–RIS link, and RIS–Bob link, respectively. $\eta_{1}∼N\left(0,\sigma_{1}^{2}I_{N}\right)$ and $\eta_{2}∼N\left(0,\sigma_{2}^{2}\right)$ are the thermal noises at RIS and Bob, respectively. Specifically, the reflecting coefficient matrix of the active RIS $\Theta_{\mathrm{Active}}=\mathrm{diag}(\phi_{1},\phi_{1},\cdots ,\phi_{N})$ is diagonal. The reflecting coefficient of the n-th reflection element at the active RIS is denoted by $\phi_{n}=a_{n}e^{j\theta_{n}},\;n=1,2,\ldots ,N$, where $a_{n}$ and $\theta_{n}$ represent the amplitude and the phase, which can be continuously varied within the intervals $\left[1,\psi_{\max}\right]$ and [0,2π), respectively. Specifically, we consider that the RIS is connected with Alice by wire/wireless channel, and the transmission between Alice and RIS is considered to be secure.

Since the passive RIS reflects the impinging signal passively, in the passive RIS-aided system, the signal that arrives at Bob is formulated as

(2) $$R_{\mathrm{Passive}}=\sqrt{p_{t}}H_{\mathrm{AB}}S+H_{\mathrm{RB}}\Theta_{\mathrm{Passive}}\sqrt{p_{t}}H_{\mathrm{AR}}S+\eta ,$$

where $\Theta_{\mathrm{Passive}}$ is a diagonal matrix as the reflection coefficient of the passive RIS and η is the thermal noise at Bob. Specifically, $\Theta_{\mathrm{Passive}}$ is a special case of $\Theta_{\mathrm{Active}}$, where $a_{n}=1$, n=1,2,…,N. Therefore, we consider the PLA design in the passive RIS-aided systems as a special case of that in the active RIS-aided systems, and denote the reflection coefficient of RIS as Θ.

As shown in Figure 2, we design an Alice–Eve difference amplifier at Alice, which controls the reflection coefficient of RIS. The proposed CR-PLA scheme jointly optimizes the communication and security performance, i.e., the achievable rate of the system and the authentication accuracy. To be more specific, the higher $a_{n}=1$, n=1,2,…,N, will increase the difference between the Alice–RIS channel estimation and Eve–RIS channel estimation. However, it will also amplify the RIS-correlated noise as well, leading to a decrease in the achievable rate of the system. Hence, the proposed CR-PLA scheme will find an optimal Θ for the joint problem. When $a_{n}=1$, i.e., passive RIS-aided PLA, the Θ is designed to maximize the achievable rate of the system. The reason is that the RFF/CF estimation suffers from the channel noises and interferences. Therefore, the authentication enhancement of RFF/CF-based PLA technique is equivalent to the improvement of communication performance based on the passive RIS.

**Figure 3:**
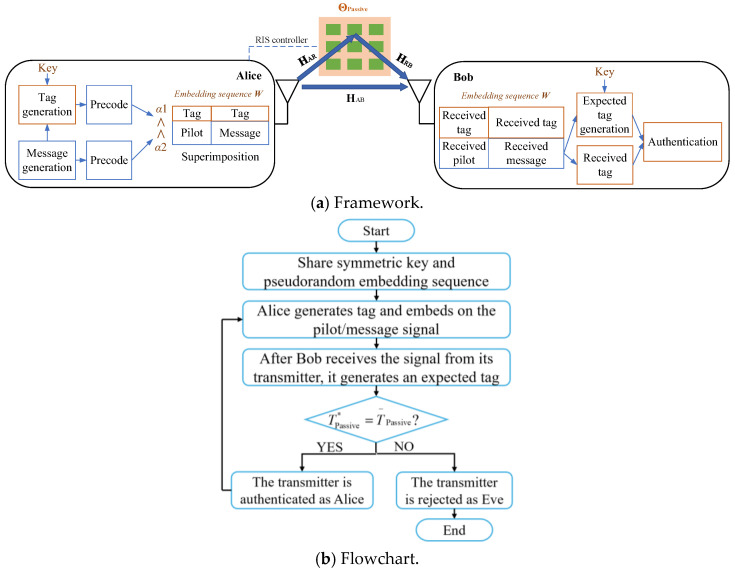
The proposed WH-PLA scheme. Bob authenticates the transmitter based on the tag embedded in the transmit signals. (**a**) Framework; (**b**) Flowchart.

*(2) Designs at Bob:* Estimates of Alice’s RFF/CF during the registration are denoted as $H_{AB}$. Then, Bob identifies the new transmitter by estimating its RFF/CF H and comparing it with $H_{AB}$. The authentication is performed as follows:

(3) $$\left\{\begin{array}{cc} H=H_{AB}, & \Phi_{1}\\ H\neq H_{AB}, & \Phi_{0} \end{array}\right.,$$

where $\Phi_{1}$ represents that this transmitter is authenticated as Alice; otherwise, it is identified as Eve, as shown in Figure 3. Again, to optimize both the communication and PLA performance, the reflecting coefficient matrix of the RIS Θ has to be well designed. On one hand, the design of Θ should balance the conflict between the received signal power maximization and the RIS-correlated noise minimization at Bob. On the other hand, it should also maximize the difference between the Alice–Bob channel and Eve–Bob channel; thus, the authentication performance relying on RFF/CF can be optimized.

*(3) Physical layer authentication enhancement:* To achieve the best trade-off between communication and authentication performance, we formulate a standard optimization problem for the CR-PLA scheme. The goal is to maximize the authentication accuracy, i.e., minimize the authentication error probability, by designing the reflection coefficient matrix Θ, while satisfying a minimum achievable rate requirement for the legitimate link.

Let $P_{\mathrm{FA}}\left(\Theta \right)$ and $P_{\mathrm{MD}}\left(\Theta \right)$ denote the false alarm and misdetection probabilities of the hypothesis test in (3), respectively. The overall authentication error probability is $P_{\mathrm{err}}\left(\Theta \right)=P_{\mathrm{FA}}\left(\Theta \right)+P_{\mathrm{MD}}\left(\Theta \right)$. The achievable rate of the Alice–Bob link is given by

(4) $${\tilde{R}}_{\mathrm{AB}}\left(\Theta \right)=\log_{2}\left(1+\mathrm{SNR}\right),$$

where SNR is the SNR of the signal that arrives at Bob. According to (1), the SNR of the signal that arrives at Bob in the active RIS-aided system can be expressed as

(5) $${\mathrm{SNR}}_{\mathrm{Active}}=\frac{p_{t}{\left|H_{AB}+H_{RB}^{†}\Theta_{\mathrm{Active}}H_{AR}\right|}^{2}}{\sigma_{2}^{2}+\sigma_{1}^{2}\sum_{n=1}^{N}{\left|H_{RB,n}\right|}^{2}a_{n}^{2}}.$$

Similarly, according to (2), the SNR of the signal that arrives at Bob in the passive RIS-aided system is given as

(6) $${\mathrm{SNR}}_{\mathrm{Passive}}=\frac{p_{t}{\left|H_{AB}+H_{RB}^{†}\Theta_{\mathrm{Passive}}H_{AR}\right|}^{2}}{\sigma_{2}^{2}}.$$

**Algorithm 1: SPSA-based projected gradient descent for CR-PLA scheme****Input:** Initial reflection coefficients $\Theta^{\left(0\right)}$ Step size μ>0 SPSA perturbation size c>0 Number of iterations K Rate constraint $R_{\min}$ Feasible set F, i.e., unit modulus for passive RIS, bounded amplitude for active RIS Smoothing parameter δ for projection
**Output:** Optimized $\Theta^{*}$
Initialize $\Theta \leftarrow \Theta^{\left(0\right)}$For k=1 to K do//Step 1: Estimate gradient of $P_{\mathrm{err}}\left(\Theta \right)$ using SPSAGenerate a random perturbation vector $\Delta \in {\mathbb{C}}^{N}$ with i.i.d. Bernoulli ±1 entries (real and imaginary parts independent).Compute perturbed points: $\Theta_{+}=\Theta +c\Delta ,\Theta_{-}=\Theta -c\Delta$Evaluate the authentication error probability $P_{\mathrm{err}}\left(\Theta_{+}\right)$ and $P_{\mathrm{err}}\left(\Theta_{-}\right)$Compute SPSA gradient estimate: $\nabla P_{\mathrm{err}}=\frac{P_{\mathrm{err}}\left(\Theta_{+}\right)-P_{\mathrm{err}}\left(\Theta_{-}\right)}{2c}\Delta^{-1}$, where $\Delta^{-1}$ denotes elementwise inverse.//Step 2: Gradient descent update $\Theta_{\mathrm{temp}}\leftarrow \Theta -\mu \nabla P_{\mathrm{err}}$//Step 3: Project onto feasible set $\Theta \leftarrow {\mathrm{Proj}}_{F}\left(\Theta_{\mathrm{temp}}\right)$
For passive RIS: project each element to the unit circle
For active RIS: project amplitude to $\left[1,\psi_{\max}\right]$ and phase to [0,2π)
10.//Step 4: Enforce rate constraint11.   $\mathrm{If}\;{\tilde{R}}_{\mathrm{AB}}\left(\Theta \right)<{\tilde{R}}_{\min}$ then12.       Reduce step size μ←μ/2 and revert Θ to previous value13.   End If14.End For15.$\mathrm{Return}\;\Theta^{*}$


Then, the optimization problem of CR-PLA is stated as:

(7) $$\begin{array}{l} \;\;\underset{\Theta }{\min}\;P_{\mathrm{err}}\left(\Theta \right)\\ s.t.\;{\tilde{R}}_{\mathrm{AB}}\left(\Theta \right)\geq {\tilde{R}}_{\min} \end{array}$$

where ${\tilde{R}}_{\min}$ is a predefined rate threshold. Since $P_{\mathrm{err}}\left(\Theta \right)$ is a non-convex function of Θ, we solve the problem by a projected gradient descent method over the manifold of (active) reflection coefficients. The gradient is computed using the simultaneous perturbation stochastic approximation (SPSA) method to avoid explicit differentiation. The constraints are handled by projection onto the feasible set after each gradient step. The detailed algorithm is summarized in Algorithm 1.

In a nutshell, the proposed CR-PLA scheme utilizes both the physical layer property, i.e., RFF/CF, and the controllable reflection of RIS. More importantly, the proposed scheme amplifies the RFF/CF estimation difference between the Alice–Bob and Eve–Bob channels. Hence, it is easier for Bob to identify Alice from Eve, achieving higher authentication accuracy.

### 3.2. WH-PLA: Watermark Hopping-Based PLA Scheme

To answer question Q2, we propose the WH-PLA scheme for passive RIS-enabled systems, where Alice embeds the tag on the pilot/message signal and transmits the weighted superposition of the pilot/message and tag signals based on a designed embedding sequence W. The detailed design of the proposed WH-PLA scheme is shown in Figure 3.

*(1) Designs at Alice:* One can observe from Figure 2 that the method of tag superimposed on pilot is easy for Bob to authenticate the transmitter without the process of source message recovery, while difficult for Eve to decode the tag, enhancing the covertness of authentication information. However, the tag embedded on the pilot signal can be seen as the interference for channel estimation and equalization of RIS-aided wireless communication systems. On the other hand, the method of tag superimposed on the message signal requires a process of message recovery before the authentication, leading to longer time latency. Hence, by designing a pseudo-random embedding sequence W in the proposed WH-PLA scheme, Alice can hop the watermarking on the pilot or message signal alternatively for better channel estimation accuracy and authentication efficiency. Moreover, it can also increase the covertness of the PLA, since it is more difficult for Eve to observe and crack the authentication method by providing extra uncertainty of security information.

The tag is generated from the message by using a symmetric key between Alice and Bob as well as a hash function. The tag generation can be expressed as

(8) $$T_{\mathrm{Passive}}=h_{\mathrm{Passive}}(M,K),$$

where $h_{\mathrm{Passive}}$ is a hash function in this scheme, M and K represent the message and key, respectively. Then, the generated tag is embedded on the pilot/message signal with weight factors $\alpha_{1}$ and $\alpha_{2}$, which satisfy $\alpha_{1}^{2}+\alpha_{2}^{2}=1,$ $\alpha_{1},\alpha_{2}\in (0,1)$, and $\alpha_{1}<<\alpha_{2}$. Hence, the transmitted signal is pressed as

(9) $$S_{\mathrm{Passive}}=\{\alpha_{1}T_{\mathrm{Passive}}+\alpha_{2}S_{P},S_{M}\}\;\mathrm{or}\;S_{\mathrm{Passive}}=\{S_{P},\alpha_{1}T_{\mathrm{Passive}}+\alpha_{2}S_{M}\}$$

in the case that the tag is superimposed on the pilot or message, respectively. $S_{M}$ and $S_{P}$ represent the message signal and pilot signal, respectively.

*(2) Designs at Bob:* Since the passive RIS reflects the impinging signal passively, the transmitted signal travels the direct link and RIS-aided link. The signal that arrives at Bob through a direct link can be expressed as

(10) $$R_{D,\mathrm{Passive}}=\sqrt{p_{t}}H_{\mathrm{AB}}S_{\mathrm{Passive}}.$$

The signal that arrives at Bob through the RIS-aided link is derived as

(11) $$R_{\mathrm{ID},\mathrm{Passive}}=\sqrt{p_{t}}H_{\mathrm{RB}}\Theta_{\mathrm{Passive}}H_{\mathrm{AR}}R_{\mathrm{Passive}}.$$

After Bob receives the signal from its transmitter, it generates an expected tag $T_{\mathrm{Passive}}^{*}$ using hash function $h_{\mathrm{Passive}}(•)$ based on the decoded message and the shared symmetric key. Then, it compares the expected tag with the tag embedded on the received signal ${\tilde{T}}_{\mathrm{Passive}}$. The authentication is performed as follows:

(12) $$\left\{\begin{array}{l} T_{\mathrm{Passive}}^{*}={\tilde{T}}_{\mathrm{Passive}},\Phi_{1}\\ T_{\mathrm{Passive}}^{*}\neq {\tilde{T}}_{\mathrm{Passive}},\Phi_{0} \end{array}\right..$$

If the expected tag is the same as the received tag, i.e., $T_{\mathrm{Passive}}^{*}={\tilde{T}}_{\mathrm{Passive}}$, the transmitter is verified to be Alice, otherwise, it is deemed to be Eve.

To achieve the best performance in both RIS-aided communication and security, the weight factors $\alpha_{1}$ and $\alpha_{2}$ as well as the reflection coefficient of RIS $\Theta_{\mathrm{Passive}}$ should be well designed in the proposed WH-PLA scheme.

(13) $$\begin{array}{l} \underset{\alpha_{1},\alpha_{2},\Theta_{\mathrm{Passive}}}{\min}P_{\mathrm{MD}}\left(\alpha_{1},\alpha_{2},\Theta_{\mathrm{Passive}}\right)\\ s.t.,\;P_{\mathrm{FA},}\leq \zeta \end{array},$$

where $P_{\mathrm{MD}}$ and $P_{\mathrm{FA}}$ represent the misdetection rate and false alarm rate of the proposed WH-PLA scheme, respectively. ζ is the threshold of false alarm rate. To be more specific, a higher $\alpha_{1}$ (i.e., lower $\alpha_{2}$) will lead to lower covertness but higher authentication accuracy. The reason is that it is more difficult for Eve to intercept and decode the tag when it is allocated with a lower power, i.e., $\alpha_{1}$. However, due to the interference and complex channel of RIS-aided wireless communications, a lower $\alpha_{1}$ will also lead to greater difficulty in comparing it with an expected tag on Bob’s side. Hence, there is a trade-off between the covertness and authentication accuracy in designing weight factors $\alpha_{1}$ and $\alpha_{2}$. On the other hand, there is a trade-off between the communication and security performance in designing $\Theta_{\mathrm{Passive}}$. The reason is that Alice should adjust the reflection coefficient of the RIS for meeting both the communication and security performance.

The design of the weight factors $\alpha_{1}$ and $\alpha_{2}$ as well as the reflection coefficient of RIS $\Theta_{\mathrm{Passive}}$ will be formulated as a joint optimization problem, which could be a nonconvex problem. In solving the joint optimization problem, we will design a hybrid learning algorithm for reaching a sub-optimal solution. The core idea of the algorithm is to decompose the non-convex joint optimization problem of (13) into two convex sub-problems by fixing one set of variables while optimizing the other, and repeat the alternating iteration until the objective function converges to a sub-optimal solution.

Firstly, the weight factors $\alpha_{1}$ and $\alpha_{2}$ are updated firstly by giving a fixed $\Theta_{\mathrm{Passive}}$. The optimization sub-problem for $\alpha_{1}$ and $\alpha_{2}$ can be transformed into a convex problem. The convex sub-problem can be solved using the gradient ascent method to obtain the optimal weight factors $\alpha_{1}^{*}$ and $\alpha_{2}^{*}$ that minimize $P_{\mathrm{MD}}\left(\alpha_{1},\alpha_{2},\Theta_{\mathrm{Passive}}\right)$. Then, optimal weight factors $\alpha_{1}^{*}$ and $\alpha_{2}^{*}$ are fixed, and $\Theta_{\mathrm{Passive}}$ is optimized to minimize $P_{\mathrm{MD}}\left(\alpha_{1}^{*},\alpha_{2}^{*},\Theta_{\mathrm{Passive}}\right)$. For each RIS element, the optimal phase shift $\Theta_{\mathrm{Passive}}$ is obtained by maximizing the signal-to-noise ratio (SNR) of the legitimate Alice–Bob channel. The hybrid learning algorithm repeats this process to find a sub-optimal solution for the joint optimization problem of the proposed WH-PLA scheme. The hybrid learning algorithm is given in Algorithm 2.
**Algorithm 2: Hybrid learning for WH-PLA scheme****Input:** Channel estimates $H_{\mathrm{AB}}$, $H_{\mathrm{AR}}$ and $H_{\mathrm{RB}}$
Transmit power $p_{t}$
False alarm threshold ζ
Maximum number of alternating iterations $\Gamma_{\max}$
Convergence tolerance ε
**Output:** Optimal power factors $\alpha_{1}^{*},\alpha_{2}^{*}$ (with $\alpha_{2}^{*}=1-\alpha_{1}^{*}$)
Optimal RIS phase shifts $\Theta^{*}=\mathrm{diag}(e^{j\theta_{1}^{*}},\ldots ,e^{j\theta_{N}^{*}})$
Initialize $\alpha_{1}^{\left(0\right)}=0.1$, $\alpha_{2}^{\left(0\right)}=0.9$, $\Theta^{\left(0\right)}=I_{N}$, iteration counter t=0Repeat:*Step A—Optimize* $\alpha^{1},\alpha^{2}$ *with fixed* $\Theta^{\left(t\right)}$  The composite channel gain is $G=H_{AB}+H_{RB}^{†}\Theta H_{AR}$  Given the derived expression for $P_{\mathrm{MD}}$
(14)$$P_{\mathrm{MD}}(\alpha_{1})=1-\exp\left(\frac{\left(\alpha_{1}p_{t}+\frac{\sigma_{n}^{2}}{2{\left|G\right|}^{2}}\right)\ln\left(\xi \right)}{\sigma_{n}^{2}/{\left|G\right|}^{2}}\right).$$
  Solve the following convex problem using gradient descent:
(15)$$\underset{\alpha_{1}\in (0,0.5)}{\min}P_{\mathrm{MD}}\left(\alpha_{1}\right)$$
  The gradient is $\frac{dP_{\mathrm{MD}}}{d\alpha_{1}}=-\frac{p_{t}\ln\left(\zeta \right)}{\sigma_{n}^{2}/{\left|G\right|}^{2}}\exp\left(\frac{\left(\alpha_{1}p_{t}+\frac{\sigma_{n}^{2}}{2{\left|G\right|}^{2}}\right)\ln\left(\zeta \right)}{\sigma_{n}^{2}/{\left|G\right|}^{2}}\right)$  Perform a few inner iterations to obtain $\alpha_{1}^{\left(t+1\right)}$. Set $\alpha_{2}^{\left(t+1\right)}=1-\alpha_{1}^{\left(t+1\right)}$.  *Step B—Optimize* Θ *with fixed* $\alpha_{1}^{\left(t+1\right)},\alpha_{2}^{\left(t+1\right)}$  Optimize Θ to maximize the SNR of the legitimate link:
(16)$$\underset{\Theta }{\max}|H_{AB}+\sum_{n=1}^{N}H_{RB,n}e^{j\theta_{n}}H_{AR,n}{|}^{2}$$
  For a passive RIS, the optimal phase shift for the n-th element is:
(17)$$\theta_{n}^{*}=\arg\left(H_{AB}^{*}\right)-\arg\left(H_{AR,n}\right)-\arg\left(H_{RB,n}\right)$$
  Set $\Theta^{\left(t+1\right)}=\mathrm{diag}\left(e^{j\theta_{1}^{*}},\ldots ,e^{j\theta_{N}^{*}}\right)$.  *Step C—Check convergence*  Compute $\Delta =\left|P_{\mathrm{MD}}\left(\alpha_{1}^{(t+1)},\Theta^{\left(t+1\right)}\right)-P_{\mathrm{MD}}\left(\alpha_{1}^{\left(t\right)},\Theta^{\left(t\right)}\right)\right|$.  If Δ<ε or $t\geq \Gamma_{\max}$, go to Step 3. Else, set t=t+1 and go to Step A.Output the final solutions: $\alpha_{1}^{*}=\alpha_{1}^{\left(t+1\right)},\alpha_{2}^{*}=1-\alpha_{1}^{*},\Theta^{*}=\Theta^{\left(t+1\right)}$

## 4. Analysis and Performance Evaluation

This section discusses and analyzes the role of RIS in the proposed schemes to answer the questions given in Section 1, i.e., Q1 and Q2. Moreover, to verify the utility of RIS for PLA and the validity of the proposed schemes, simulation results are provided. Specifically, the simulation parameters are shown in Table 3.

### 4.1. The CR-PLA Scheme

In the RFF/CF-based PLA technique, the RFF or CF features are utilized for authentication, which suffer from the inaccurate estimation in dynamic wireless communications. The main challenge of this technique is to utilize the imperfectly estimated and time-varying feature estimation to achieve accurate authentication. As it can be observed in the proposed CR-PLA scheme (i.e., Figure 3), the passive/active RIS is used to increase the RFF/CF estimation accuracy through increasing the SNR of the wireless communication systems. More importantly, the RFF/CF estimations between Alice and Eve can be amplified through controllable reflection design at RIS. Hence, the proposed CR-PLA scheme enhances authentication performance with the assistance of RIS.

**Figure 4:**
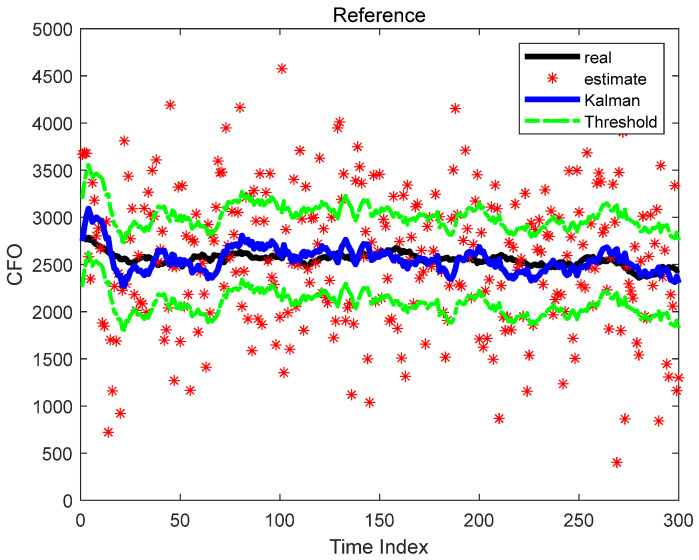
The CFO estimations without RIS.

**Figure 5:**
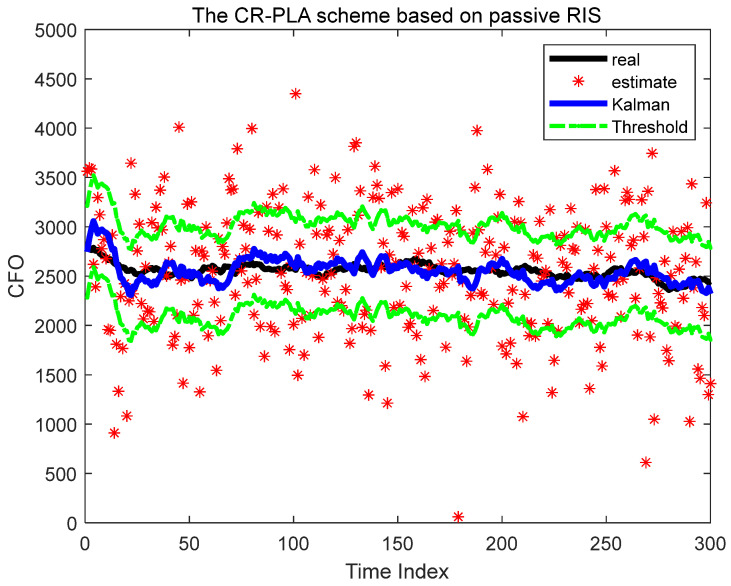
The CFO estimations based on the proposed CR-PLA scheme in a passive RIS-aided system.

**Figure 6:**
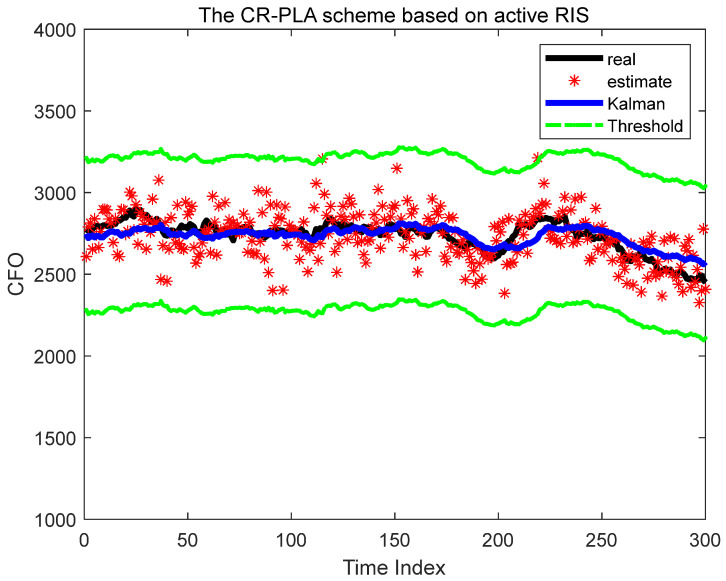
The CFO estimations based on the proposed CR-PLA scheme in an active RIS-aided system with amplified power of 15 dB.

Figure 4, Figure 5 and Figure 6 characterize the comparison results of the CFO estimations based on the proposed CR-PLA scheme utilizing both passive and active RIS. We utilize the Kalman filter-based predictor and the authentication threshold of [7] in the simulation. It can be observed from Figure 4 that the system without RIS suffers from a low authentication accuracy, i.e., many CFO estimates are out of the authentication threshold range, due to the channel noise and inaccurate estimation of Alice’s CFO.

It can be observed from Figure 5 and Figure 6 that, compared with the CFO estimation without the assistance of RIS, the CFO estimations of the proposed CR-PLA scheme utilizing passive/active RIS are more accurate. Moreover, the CFO estimations of the proposed CR-PLA scheme utilizing active RIS are the best. The reason is that the utilization of both passive RIS and active RIS increases the SNR of the communication system, which will benefit the CFO estimation. A higher SNR effectively mitigates the interference and noise impairment, thereby further improving the accuracy and robustness of CFO estimation, as well as improving the authentication performance. Essentially, the CFO estimation accuracy directly determines the authentication accuracy in this framework. Higher CFO estimation accuracy contributes to more reliable feature extraction, and thus further improves the overall authentication performance.

**Figure 7:**
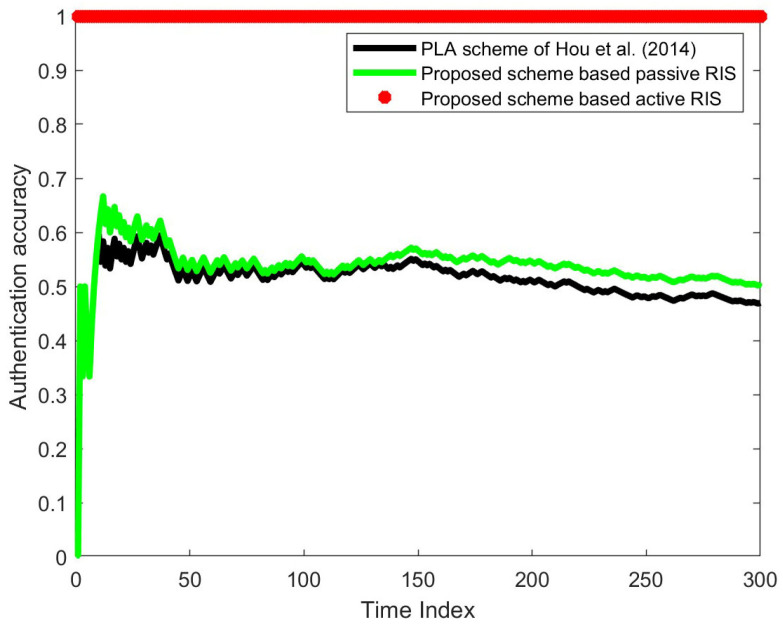
Authentication accuracy comparison among the PLA scheme of [7] and the proposed passive RIS-based and active RIS-based CR-PLA schemes.

It can be observed from Figure 7 that the proposed active RIS-based CR-PLA scheme consistently achieves the highest authentication accuracy across the entire time horizon, followed by the proposed passive RIS-based CR-PLA scheme. The PLA scheme of [7] exhibits the lowest accuracy. The results of this figure demonstrate the advantage of using RIS in physical layer authentication enhancement.

### 4.2. The WH-PLA Scheme

The WH-PLA scheme is proposed for RIS-enabled wireless communication systems by designing a pseudorandom embedding sequence. The authentication tags are embedded on the pilot or message signals alternatively for higher uncertainty and randomness, thus enhancing security. In this scheme, weight factors $\alpha_{1}$ and $\alpha_{2}$ as well as the reflection coefficient of RIS $\Theta_{\mathrm{Passive}}$ are required to be optimally designed for better performance, including both the security and communication performance. The RIS is used in the proposed WH-PLA scheme for joint security and communication enhancement, i.e., the improvement of trade-off between the authentication accuracy and SNR of communication system.

**Figure 8:**
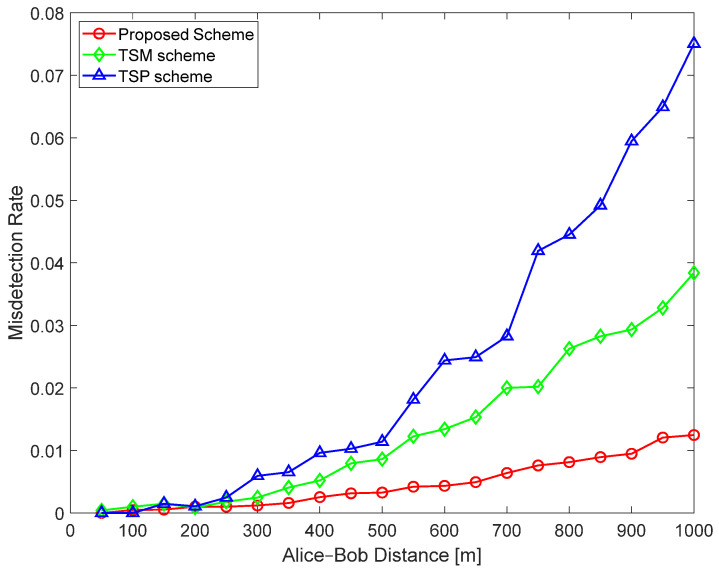
Misdetection rate of the proposed WH-PLA scheme vs. the distance between Alice and Bob.

Figure 8 characterizes the misdetection rate of the proposed WH-PLA scheme compared with the tag superimposed on the message (TSM) scheme of [13] and the tag superimposed on the pilot (TSP) scheme of [14]. It can be observed from this figure that the misdetection rate of the proposed WH-PLA scheme is much lower than both the TSM scheme and the TSP scheme. The reason is that the proposed WH-PLA scheme increases the randomness of authentication information superimposed on the transmit signals by designing a pseudo-random embedding sequence. It is more difficult for Eve to decode both the authenticated tag superimposed on the message/pilot signal and the pseudo-random embedding sequence. Moreover, the misdetection rate of the TSM scheme is lower than the TSP scheme. The reason is that the length of authentication tag in the TSM scheme is longer than that in the TSP scheme, and the tag generation strategy in the TSM based on both the message and key provides higher randomness for authentication than that in the TSP scheme.

**Figure 9:**
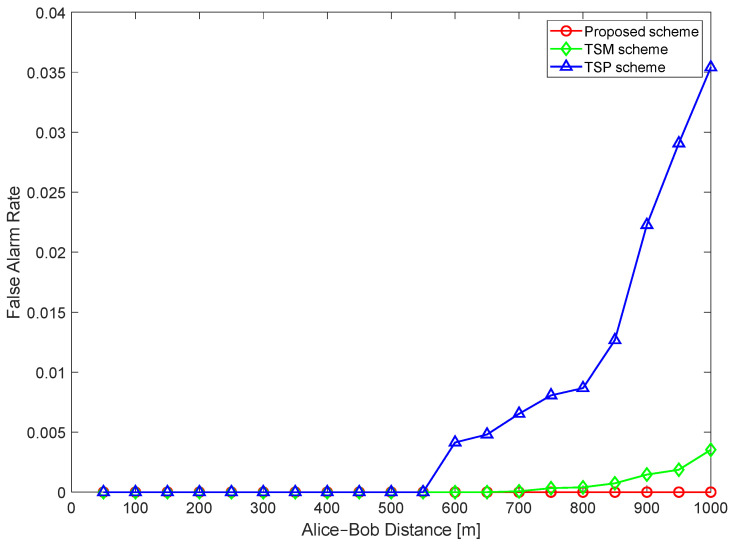
False alarm rate of the proposed WH-PLA scheme vs. the distance between Alice and Bob.

Figure 9 compares the false alarm rate of the proposed WH-PLA scheme with the TSM scheme in [13] and the TSP scheme in [14]. The results show that WH-PLA achieves a substantially lower false alarm rate than both benchmarks. This advantage stems from the pseudo-random embedding sequence adopted in WH-PLA, which enhances the randomness of the superimposed authentication information and adds more authentication information. In addition, the TSM scheme outperforms the TSP scheme in false alarm rate, since the TSM scheme employs a longer authentication tag and delivers more authentication information.

## 5. Challenges and Open Research Issues

Introducing the RIS to PLA provides a promising and flexible method for security enhancement, as well as could bring many benefits by utilizing the controllable reflection of RIS. By leveraging the channel properties of the RIS-aided wireless communications, the proposed schemes enhance security based on the RFF/CF estimations and the watermarking embedded on the signals, which are highly random and difficult for third parties to crack. Moreover, the proposed schemes do not require high frequency of renewing the symmetric key to guarantee the security level, which dramatically reduces the computation and communication overhead of generating, renewing, and distributing keys.

There are still some challenges and open issues to be solved:

(1) *Trade-off between the communication and security:* Provisioning the security for the RIS-aided system will decrease the communication performance, since the reflecting coefficient matrix of the RIS is designed for jointly optimizing both the communication and security performance. How to improve the trade-off will be an open issue for better PLA design in RIS-aided systems. Moreover, as discussed above, the problems of PLA in RIS-aided systems could be nonconvex, which will be very difficult to find an optimal solution. Hence, how to solve the designed nonconvex optimization problem will be very challenging.
(2) *Difference between uplink and downlink of RIS-aided systems:* This makes the designs of PLA more challenging for mutual authentication since the RIS is not controlled by both transmitter and receiver in most of the cases. In the proposed schemes, only Alice controls the RIS, and the authentication of uplink from Bob to Alice requires different designs. Specifically, the proposed CR-PLA scheme relies on the control of RIS for authentication, which is not suitable for the uplink authentication if Bob cannot control the amplification. Moreover, the designs will be more difficult in the frequency division duplex (FDD) systems.
(3) *RIS attacks encountered:* In the above PLA designs, we assume that the RIS is controlled by Alice and is legitimate. However, the RIS may be attacked and controlled by Eve in some scenarios. Hence, the PLA designs in RIS-aided systems could be more challenging to ensure the legitimacy of both transmitter and RIS.

## 6. Conclusions

This article introduced the RIS to PLA designs by fully utilizing the channel properties and the controllable reflection at the physical layer. Two schemes were proposed for both passive and active RIS-aided systems, namely for CR-PLA and WH-PLA, respectively. To be more specific, the controllable reflection of active RIS was designed for improved communication performance and authentication accuracy at the physical layer in the CR-PLA scheme by increasing the RFF/CF estimation accuracy. The watermark hopping was designed in the WH-PLA scheme to embed the authentication tag on the pilot signal or message signal alternatively. The power allocation of tag and pilot/message was also designed for the increase in trade-off between security and communication. The existing challenges and open research issues were also introduced for future considerations.

## Figures and Tables

**Table 1:** Comparisons of different PLA techniques, including the radio frequency fingerprint (RFF)-based, channel fingerprint (CF)-based, and tag embedding (TE)-based techniques.

Schemes	Channel Estimation	Source Message Recovery	SNR	Spectral Efficiency	Covertness	Advantages	Challenges
CFO-based PLA [7]	√	×	-	-	High	Hardware-dependent, difficult to imitate and predict	Minor difference among multiple devices, difficult to estimate accurate RFF
I/Q imbalance-based PLA [8]	√	×	-	-	High		
CIR/CFR-based PLA [9]	√	×	-	-	High	Channel, environment, location-dependent	Dynamic and inaccurate estimations of CF
RSSI-based PLA [10]	×	×	-	-	Low		
Encoding of pilot/message [11]	√	√	-	-	Low	Controllable security information embedding	Trade-off between communication and authentication performance
Tag multiplied in message [12]	√	√	↓	↓	High		
Tag superimposed on message signal [13]	√	√	↓	↓	High		
Tag superimposed on pilot signal [14]	√	×	-	↓	High		

**Table 2:** Symbols used in this paper.

Symbol	Definition	Symbol	Definition
N	Number of reflecting elements of RIS	$H_{\mathrm{AB}}$	Channel gain of Alice–Bob link
$H_{\mathrm{AR}}$	Channel gain of Alice–RIS link	$H_{\mathrm{RB}}$	Channel gain of RIS–Bob link
$H_{\mathrm{EB}}$	Channel gain of Eve–Bob link	$H_{\mathrm{ER}}$	Channel gain of Eve–RIS link
Θ	Reflecting coefficient matrix of RIS	$\Theta_{\mathrm{Active}}$	Reflecting coefficient matrix of an active RIS
$\Theta_{\mathrm{Passive}}$	Reflecting coefficient matrix of a passive RIS	${\tilde{R}}_{\mathrm{AB}}$	Achievable rate of the Alice–Bob link
$p_{t}$	Transmit power of Alice	$\eta_{1}$	Thermal noises at RIS
$\eta_{2}$	Thermal noises at Bob	R	Signal arrives at Bob
SNR	SNR of the signal arrives at Bob	S	Transmit signal
$P_{\mathrm{FA}}$	False alarm rate	$P_{\mathrm{MD}}$	Misdetection rate
$\Phi_{1}$	Case that this transmitter is authenticated as Alice	$\Phi_{0}$	Case that this transmitter is authenticated as Eve
$H_{AB}$	Estimate of Alice’s RFF/CF during the registration	H	New estimate of the transmitter’s RFF/CF

**Table 3:** Simulation parameters.

Parameter	Value	Parameter	Value
Carrier frequency	3.5 GHz	Bandwidth	25 MHz
Sampling rate	25 MHz	Transmit power	20 dBm
Path loss exponent	3.5	Reference distance	1 m
RIS element count	32	Active RIS amplification	5
Noise figure	5 dB	False alarm threshold	0.05

## Data Availability

The raw data supporting the conclusions of this article will be made available by the authors upon request.
